# Primary prevention of cardiovascular diseases: a cost study in family practices

**DOI:** 10.1186/1471-2296-12-69

**Published:** 2011-07-06

**Authors:** Esther W de Bekker-Grob, Sandra van Dulmen, Matthijs van den Berg, Robert A Verheij, Laurentius CJ Slobbe

**Affiliations:** 1 Department of Public Health, Erasmus MC - University Medical Centre Rotterdam, (Dr. Molewaterplein 50), Rotterdam, (3000 CA), The Netherlands; 2 Netherlands Institute of Health Services Research, (Otterstraat 118-124), Utrecht, (3513 CR), The, Netherlands; 3 National Institute of Public Health and the Environment, Centre for Public Health Forecasting, (Antonie van Leeuwenhoeklaan 9), Bilthoven, (3721 MA), The Netherlands

## Abstract

**Background:**

Considering the scarcity of health care resources and the high costs associated with cardiovascular diseases, we investigated the spending on cardiovascular primary preventive activities and the prescribing behaviour of primary preventive cardiovascular medication (PPCM) in Dutch family practices (FPs).

**Methods:**

A mixed methods design was used, which consisted of a questionnaire (n = 80 FPs), video recordings of hypertension- or cholesterol-related general practitioner visits (n = 56), and the database of Netherlands Information Network of General Practice (n = 45 FPs; n = 157,137 patients). The questionnaire and video recordings were used to determine the average frequency and time spent on cardiovascular primary preventive activities per FP respectively. Taking into account the annual income and full time equivalents of general practitioners, health care assistants, and practice nurses as well as the practice costs, the total spending on cardiovascular primary preventive activities in Dutch FPs was calculated. The database of Netherlands Information Network of General Practice was used to determine the prescribing behaviour in Dutch FPs by conducting multilevel regression models and adjusting for patient and practice characteristics.

**Results:**

Total expenditure on cardiovascular primary preventive activities in FPs in 2009 was €38.8 million (€2.35 per capita), of which 47% was spent on blood pressure measurements, 26% on cardiovascular risk profiling, and 11% on lifestyle counselling. Fifteen percent (€11 per capita) of all cardiovascular medication prescribed in FPs was a PPCM. FPs differed greatly on prescription of PPCM (odds ratio of 3.1).

**Conclusions:**

Total costs of cardiovascular primary preventive activities in FPs such as blood pressure measurements and lifestyle counselling are relatively low compared to the costs of PPCM. There is considerable heterogeneity in prescribing behaviour of PPCM between FPs. Further research is needed to determine whether such large differences in prescription rates are justified. Striving for an optimal use of cardiovascular primary preventive activities might lead to similar health outcomes, but may achieve important cost savings.

## Background

Cardiovascular diseases (CVDs) are the leading cause of death and a major cause of disability and loss of productivity in adults world wide [1]. The annual cost of CVD is estimated to be €169 billion a year in the enlarged European Union and $394 billion (€296) in the USA [2,3]. CVD is generally caused by a combination of several risk factors such as smoking, high blood cholesterol, high blood pressure, physical inactivity, obesity and overweight. Risk factor modification has been unequivocally shown to reduce mortality and morbidity in people with or without established CVD [4].

Family practices (FPs) can play an important role in risk factor modification for people with an increased risk of CVD. The general practitioner, and the practice nurse and the health care assistant to some extent, can reduce the risk of CVD by conducting cardiovascular preventive activities (cardiovascular primary preventive activities are preventive activities (e.g. prescribing of blood-pressure-lowering drugs, or lifestyle counselling) aimed at a *determinant *of cardiovascular disease for people without CVD) [5-9].

As the population ages the annual costs of CVD are likely to increase. Considering the scarcity of health care resources and the high costs of CVD [10], a comprehensive overview of spending on cardiovascular preventive activities in FPs is crucial for health policy development and evaluation. However, such an overview of spending on cardiovascular primary preventive activities in FPs is lacking [11]. Moreover, information is lacking on the proportion of cardiovascular medication prescriptions that are for primary prevention (i.e., cardiovascular medication aimed at a determinant of cardiovascular disease for people with neither cardiovascular nor diabetes or lipid disorders), and how much general practitioners differ in prescribing behaviour regarding primary preventive cardiovascular medication. Insights into these issues are important as the amounts spent on cardiovascular medication (predominantly statins and antihypertensive medication) are considerable [11], but in case of primary prevention may not always be strictly needed. The Dutch multidisciplinary guideline for cardiovascular risk management recommends that people without a history of CVD, diabetes, or lipid disorder should receive primary prevention interventions such as lifestyle recommendations, blood pressure measurements or blood tests if there is a positive family history, clear overweight, or a patient's request [12]. This guideline recommends cardiovascular risk profiling if (i) the systolic blood pressure (SBP) is 140 mmHg or higher, (ii) the total cholesterol (TC) is 6.5 mmol/l or higher, or (iii) if the combination age (men ≥ 50 year, women ≥ 55 year) and smoking exists. The decision to prescribe medications for people without a history of CVD, diabetes or lipid disorders depends not only on the estimated risk of CVD, the SBP, and 'TC/high density lipoprotein cholesterol'-ratio, but also on patient's preferences [12].

This study estimates the costs of cardiovascular primary preventive activities including prescription of primary preventive cardiovascular medication (PPCM) in FPs in the Netherlands. This makes it possible to investigate i) what is done in FPs to prevent CVD in people with increased risk of CVD, ii) what the total direct medical costs are, iii) the proportion of cardiovascular medication prescriptions that are for primary prevention (to separate prevention from care), and iv) how much FPs differ in prescribing behaviour of PPCM.

## Methods

We used a mixed methods design, which consisted of three parts: i) a questionnaire among family practices (FPs); ii) video recordings of hypertension-, cholesterol- and/or endocrine-related general practitioner visits; and iii) the database of Netherlands Information Network of General Practice. There was an overlap in the FPs used for the three different approaches.

### Questionnaire

Literature [12,13] and interviews with general practitioners (n = 3) were used to determine cardiovascular primary preventive activities for patients without CVD. Six activities were selected for the questionnaire: 1) blood pressure measurement; 2) cardiovascular risk profiling; 3) a blood test related activity (i.e., explaining the need for a blood test to the patient, filling out a request for a blood test, and/or sharing the blood test results with the patient); 4) family history; 5) lifestyle history; and 6) lifestyle counselling. The questionnaire comprised questions on practice size and location, and about the frequency of the above mentioned cardiovascular primary preventive activities per week provided by general practitioners, health care assistants and practice nurses.

The questionnaire was emailed to 80 FPs from the **'**Netherlands Information Network of General Practice' (LINH-DB) [14,15] in 2009. Each year LINH contains 80 FPs and is a representative network of Dutch FPs as it takes into account the representativeness regarding practice type, urbanisation and the software system that is used [16]. The sample of practices originates from the mid 1990's; eighty FPs are sufficient to make disease-related conclusions at the Dutch national level. Practices participate on a voluntary basis. The LINH database holds longitudinal data on morbidity, prescribing, and referrals, based on the routine electronic patient records that are kept by the participating practices. In order to enable longitudinal analyses, changes in the set of participating practices are kept to a minimum. There is a waiting list for practices to participate in the network. When a practice quits participating in the network, a new practice is invited. These FPs are spread throughout the Netherlands and are representative of all Dutch FPs. Reminders were sent to non-respondents two and four weeks later.

### Video recordings

We used video recorded general practitioner consultations which were recorded as part of a larger study into doctor-patient communication in general practice in 2007-2008 [17]. Neither general practitioners nor patients were aware of the topics of interest for the researchers. Forty Dutch general practitioners participated in the study. These general practitioners are representative of Dutch general practitioners regarding age, practice form and number of days worked [17]. Eight hundred and eight consultations were video-recorded. These consultations were randomly recorded on week days, and are expected to represent Dutch general practice consultations. The study protocol adheres to the Dutch privacy legislation, approved by the Dutch Data Protection Authority. However, approval by a medical ethics committee was not required for this observational study, because the study did not interfere with a GPs usual work process and patients were not confronted with whatever project-related intervention. Our research complied with the Helsinki Declaration. All participating general practitioners and patients filled in an informed consent form before the recording of the consultation.

We included all recorded hypertension-, cholesterol- and endocrine-related visits for further analyses (we assumed that, for example, the time spent on a blood pressure measurement did not differ between primary and secondary prevention). The time spent on any of the six cardiovascular preventive activities (see previous paragraph) was measured.

### Database of Netherlands Information Network of General Practice

Data on diagnosis and prescriptions were derived from the 'Netherlands Information Network of General Practice' (LINH-DB) [14,15]. Focusing on the period 2005-2007, data from 161 FPs, spread throughout the Netherlands, were collected (data on the periods 2008 and 2009 were not yet available at the time of study). To investigate the prescription of primary preventive cardiovascular medication (PPCM) in FPs, we included FPs with complete data sets over the whole period 2005-2007. PPCM is defined as cardiovascular medication (i.e., all kind of beta blockers and statins) aimed at a determinant of cardiovascular disease for people without cardiovascular diseases, diabetes (types 1 and 2), or disorders of lipid metabolism (e.g. hypercholesterolemia). Cardiovascular treatment of patients who had consulted their general practitioner for cardiovascular diseases, diabetes, or lipid disorders in 2005-2007 was not regarded of primary preventive nature. Patients with these conditions were excluded, because the focus of the manuscript is on primary prevention. Primary prevention focuses on patients without cardiovascular diseases and/or diabetes and/or disorders of lipid metabolism, because in that case - according the Dutch guidelines - the use of measures belongs to regular care.

### Data analysis

Based on the questionnaire, we determined the average weekly frequency a FP carried out the different cardiovascular primary preventive activities. The averages were determined separately for the general practitioners, health care assistants, and practice nurses in each practice. The video recorded consultations were used to determine the average amount of time required to carry out each of these activities. Based on these two figures we determined the average amount of time spent carrying out cardiovascular primary prevention activities per week. This was extrapolated to an annual figure. Finally, we estimated the total direct costs of cardiovascular primary preventive activities in FPs in the Netherlands in 2009 by taking into account the annual income and full time equivalents of general practitioners, health care assistants, and practice nurses as well as practice costs (i.e. housing, medical equipment, insurance and transportation) [18-20].

The volume of prescriptions of PPCM in FPs was determined using data of FPs with complete data sets from the period 2005 through 2007. Second, to get insight into which part of prescription of cardiovascular medication was intended as preventive, the patients were divided into four patient groups: 1) patients with cardiovascular and diabetes (types 1 and 2) and/or disorders of lipid metabolism (e.g. hypercholesterolemia); 2) patients with cardiovascular disease, but without diabetes or lipid disorders; 3) patients without cardiovascular disease, but with diabetes or lipid disorders; and 4) patients with neither cardiovascular disease nor diabetes or lipid disorders (primary prevention). The proportion of patients with i) prescription of cardiovascular medication, and ii) prescription of PPCM were calculated based on the existence of CVD, diabetes or lipid disordersas previously outlined. Multilevel analyses were conducted to investigate whether differences in prescription of cardiovascular medication for each patient group could be explained by family practice characteristics (urbanisation and practice type) and/or by patients' characteristics (age, gender, living in a disadvantaged neighbourhood (i.e. a geographically localised community within a larger city, town or suburb that contained a large proportion of people with a low social economic status), and insurance type). This was achieved using a mixed effects regression model, which estimates 'fixed' coefficients β for covariates at the patient level and at the family practice level i in FP j (X_ij_) and 'random' coefficients for the FPs j (θ_j_). The parameter θ_j _is assumed to be normally distributed with mean μ and variance τ^2^:

The variance (τ^2^) estimated in the model is a measure of the between family practice differences, and indicates the spreading of the prescribing behaviour of the individual FPs. To facilitate interpretation of the estimated between-FPs differences, we compared the FPs at the higher end of the outcome distribution (the 90^th ^percentile) with the FPs at the lower end (the 10^th ^percentile) of the outcome distribution. The relative difference in odds of prescribing behaviour in these two groups of FPs can be calculated from the parameter τ^2^: 80% OR range = exp (2.58*τ). The value 2.58 is the z-value corresponding to the width of the 80% (i.e. from the 10^th ^percentile to the 90^th ^percentile) confidence interval in a normal distribution (2*1.29).

## Results

### Frequency of primary preventive activities

**Figure 1:**
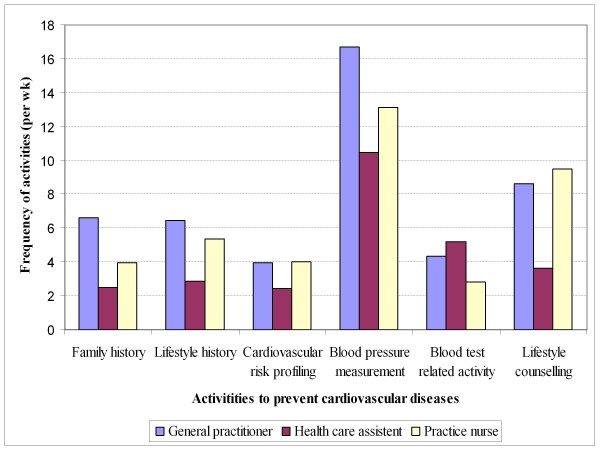
**Frequency of primary preventive activities in family practice to prevent cardiovascular diseases in the Netherlands in 2009, in amount per week per discipline**.

The response rate of FPs for the questionnaire was 85% (68/80). The average frequency of each identified cardiovascular primary preventive activity in FPs varied according to the activity and the discipline (Figure 1). Blood pressure measurement was the most frequently reported activity: 16.7, 13.1, and 10.5 times per week for a general practitioner, a practice nurse, and a health care assistant respectively. General practitioners, practice nurses, and health care assistants were involved in each of the six cardiovascular primary preventive activities.

### Time spent on primary preventive activities

Seven percent (n = 56) of the 808 video-recorded consultations were hypertension-, cholesterol- and endocrine-related. There were on average 1.7 hypertension-, cholesterol- and endocrine-related visits per general practitioner (SD 1.0; range 1-4). Cardiovascular risk profiling had the longest mean duration, whereas the family/lifestyle history had the shortest mean duration (244 and 24 seconds respectively) (Table 1). Additional File 1: Appendix shows the time spent in minutes per week per discipline on primary preventive activities in family practice to prevent cardiovascular diseases (appendix 1).

**Table 1:** Duration of primary preventive activities in family practice to prevent cardiovascular diseases in seconds (based on n = 56 video-taped general practice visits in 2007/2008)

	Duration	
	
	Mean	(SD)
Family history	23.7	(13.8)
Lifestyle history	24.1	(21.7)
Cardiovascular risk profiling	244.0	(172.5)
Blood pressure measurement	105.7	(54.8)
Blood test related activity	71.1	(22.2)
Lifestyle counselling	46.1	(47.1)

### Costs

Total expenditure on cardiovascular primary preventive activities in FPs was €38.8 million or €2.35 per capita in the Netherlands in 2009, of which 39.8% were practice costs (€15.4 million) and 60.2% were personnel costs (€23.3 million) (Table 2). The vast majority of the personnel costs were made by general practitioners (€18.6 million; 79.8%). Forty-seven percent (€1.11 per capita) of the total expenditure was related to blood pressure measurements, 26% to cardiovascular risk profiling (€0.60 per capita), and 11% to lifestyle counselling (€0.25 per capita). Approximately €181 million (i.e. 15% of the total costs of cardiovascular medication [21]) or €11 per capita was spent on PPCM (see next paragraph for more details).

**Table 2:** Estimated national spending in family practices to prevent cardiovascular diseases in the Netherlands in 2009

	Personnel costs			Practice costs	Total costs	Costs per	Portion total
					
	GP	HCA	PN			patient	costs
	
	(€)	(€)	(€)	(€)	(€)	(€)	(%)
Family history	777,496	87,028	35,761	719,706	1,619,991	0.10	4.2
Lifestyle history	772,581	101,909	49,509	685,751	1,609,749	0.10	4.2
Cardiovascular risk profiling	4,780,739	870,361	374,741	3,935,307	9,961,147	0.60	25.7
Blood pressure measurement	8,747,392	1,636,260	530,036	7,312,378	18,226,067	1.11	47.0
Blood test related activity	1,532,813	544,058	76,046	1,040,853	3,193,769	0.19	8.2
Lifestyle counselling	1,988,230	246,905	167, 852	1,739,688	4,142,675	0.25	10.7
**Total**	**18,599,251**	**3,486,520**	**1,233,945**	**15,433,683**	**38,753,399**	**2.35**	**100.0**

GP = general practitioner; HCA = health care assistant; PN = practice nurse.

### Prescription of cardiovascular medication in FPs

Forty-five out of 161 FPs (28%; n = 157,137 patients) from the 'Netherlands Information Network of General Practice' had complete data sets over the whole period 2005-2007, and were included for further analysis. The prescription of cardiovascular medication differed greatly between FPs for all four patient groups (Table 3). On average 19.2% (30,275/157,137) of the patients of the FPs had a prescription for cardiovascular medication; the prescription rate for cardiovascular medication varied between 12.0% and 27.0% for the FP with the lowest and highest prescription rates, respectively. Fifteen percent (4,543/30,275) of all cardiovascular medication prescribed in FPs was a PPCM.

**Table 3:** Prescription of cardiovascular medication in family practices in the Netherlands in 2007 (n = 45 family practices; n = 157,137 patients)

		in 2005-2007		Prescription cardiovascular medication in 2007				
		
Patient group**(n)		Cardio-vascular disease	Diabetes or lipid disorders	Lowest* (%)	Highest* (%)	Median (%)	Mean (%; n)	
1	(9,954)	Yes	Yes	75.5	95.6	87.2	87.3	(8,685)
2	(20,976)	Yes	No	49.5	77.2	65.2	64.4	(13,720)
3	(9,287)	No	Yes	15.8	56.1	36.0	36.3	(3,327)
4	(116,920)	No	No	1.0	9.3	3.5	3.9	(4,543)
Total	(157,137)			12.0	27.0	19.4	19.2	(30,275)

Notes:

* lowest = family practice with the lowest prescription rate; highest = family practice with the highest prescription rate.

** patient group 1 = patients with cardiovascular and diabetes (types 1 and 2) and/or disorders of lipid metabolism (e.g. hypercholesterolemia); patient group 2 = patients with cardiovascular disease, but without diabetes or lipid disorders; patient group 3 = patients without cardiovascular disease, but with diabetes or lipid disorders; and patient group 4 = patients with neither cardiovascular nor diabetes or lipid disorders (primary prevention).

The results of the multilevel analyses showed that the difference between FPs in prescription of cardiovascular medication for each patient group was partly explained by family practice and patient characteristics. In other words, for each patient group the value of τ^2 ^decreased when practice and/or patient characteristics were taken into account (Table 4). However, although adjusted for practice and patient characteristics, the odds range indicates considerable variation in prescribing behaviour between practices. Focusing on the patients 'without cardiovascular, diabetes and lipid disorders' (i.e. patient group 4), the between practice differences in outcome were 3.1 fold. Hence, although adjusted for practice and patient characteristics, the odds on PPCM in practices at the higher end of the outcome distribution (odds = 1.75; Table 4) was 3.1 times higher than in practices at the lower end (odds = 0.57; Table 4). Adjusted for practice and patient characteristics, 10 out of 45 FPs (22.2%) had a significantly lower prescription rate of PPCM, and 15 out of 45 FPs (33%) had a significantly higher prescription rate compared to the average prescription rate of all FPs. The coefficients of the random effects logistic regression model indicated that the probability of receiving a prescription for a cardiovascular medication for patient group 4 was significantly higher for older patients and for females, and lower for patients with private insurance and for duo FPs (Table 5). The urbanization level of the FP as well as the neighbourhood of the patient did not significantly influence the PPCM prescribing behaviour.

**Table 4:** Differences in prescribing behaviour of cardiovascular medication between family practices (n = 45; n = 157,137 patients) with unadjusted random effect estimates, adjusted random effects estimates for family practice characteristics (i.e. urbanization and practice type), random effects estimates for patient characteristics (i.e. age, gender, social economic status and insurance type), and adjusted for practice characteristics *and *patient characteristics

	**τ**^**2**^	OR range
**Patients with CVD and endocrine diseases (n = 9,954)**		
Unadjusted	0.15	0.61 - 1.65
Adjusted for FP characteristics only	0.13	0.63 - 1.59
Adjusted for patient characteristics only	0.14	0.62 - 1.62
Adjusted for FP and patient characteristics	0.12	0.64 - 1.56

**Patients with CVD (n = 20,976)**		
Unadjusted	0.6	0.73 - 1.36
Adjusted for FP characteristics only	0.5	0.76 - 1.32
Adjusted for patient characteristics only	0.5	0.75 - 1.33
Adjusted for FP and patient characteristics	0.4	0.77 - 1.29

**Patients with endocrine diseases (n = 9,287)**		
Unadjusted	0.13	0.63 - 1.58
Adjusted for FP characteristics only	0.11	0.65 - 1.53
Adjusted for patient characteristics only	0.10	0.67 - 1.50
Adjusted for FP and patient characteristics	0.08	0.69 - 1.44

**Patients without CVD and endocrine diseases (n = 116,920)**		
Unadjusted	0.29	0.50 - 2.00
Adjusted for FP characteristics only	0.25	0.52 - 1.91
Adjusted for patient characteristics only	0.23	0.54 - 1.86
Adjusted for FP and patient characteristics	0.19	0.57 - 1.75

Notes: 1) OR = odds ratio; the OR range is calculated as the range between exp(-1.29*τ) and exp(1.29*τ);

2) FP = family practice; 3) The variance τ^2 ^estimated in the random effects model is a measure of the between-FPs differences, and indicated the spreading of prescribing behaviour of the individual FPs; 4) CVD = cardiovascular disease.

**Table 5:** Influence of patient and family practice characteristics on primary preventive cardiovascular prescribing behaviour (n = 45 family practices; n = 116,920 patients)

		Random effect Adjusted	SE	p-value
Patient characteristics	Age (per 10 year)	0.512	0.009	< 0.001
	Gender (female vs male)	0.191	0.032	< 0.001
	Type of insurance (private vs national)	-0.158	0.035	< 0.001
	Disadvantage neighbourhood (yes vs no)	0.140	0.088	0.11

FP characteristics	Practice type (ref. single handed)			
	Duo	-0.540	0.214	0.01
	Group	-0.127	0.184	0.49
	Health care center	-0.133	0.255	0.60
	Urbanisation (ref. very strongly)			
	Strongly	0.249	0.237	0.29
	Moderately	-0.126	0.221	0.57
	Weakly	0.001	0.219	0.99
	not	0.226	0.193	0.24

Note: Values are random effect logistic regression coefficients. A positive number means a higher probability on prescription of cardiovascular medication.

## Discussion

This study gave a comprehensive overview of spending on cardiovascular primary preventive activities in FPs, which is quite innovative and crucial for health policy development and evaluation. Additionally, this study contributed to the literature on the variation on cardiovascular medication prescriptions; especially on primary preventive cardiovascular medication (PPCM). We found that in family practices (FPs) €38.8 million or €2.35 per capita was spent on cardiovascular primary preventive activities such as taking a family and lifestyle history, cardiovascular risk profiling, blood pressure measurement, blood test related activity, and lifestyle counselling in the Netherlands in 2009. General practitioners, health care assistants, and practice nurses were all involved in these primary preventive activities, from which blood pressure measurement was the most frequently conducted cardiovascular primary preventive activity. Nineteen percent of the patients of FPs had a prescription for cardiovascular medication. Fifteen percent of these prescriptions (approximately €181 million or €11 per capita at a national level) were primarily a preventive one. Family practices differed considerably with respect to prescription rates of primary preventive cardiovascular medication (PPCM).

To the best of our knowledge, there are no previous studies on the costs of cardiovascular primary preventive activities in FPs to compare our outcomes with. Furthermore, there are no previous studies establishing the volume of prescription of PPCM in family practices. However, in a CVD prevention context, Pelletier-Fleury et al. (2007) showed that there was a high between-doctor variability of CVD prevention in FPs [22]. This is in line with our study, which showed that there was a substantial difference in prescription of preventive cardiovascular medication between FPs. We could compare the proportion of patients with a prescription of a CVD medication, based on our data, with the proportion of patients with a prescription of a CVD medication based on the Drug Information System of the Health Care Insurance Board (GIP; http://www.gipdatabank.nl/). According to the GIP database, 20% of the Dutch population used CVD medication in 2007. This was quite similar to our study results, which showed that 19.2% of the patients had CVD medication. This suggests that the practices in our study were largely representative for all practices in the Netherlands.

The substantial differences in prescription of PPCM in FPs can partly be explained by the various combinations of patient characteristics per FP as the appropriateness of prescribing should be based on absolute cardiovascular risk. However, prescribing behaviour may also play a role in the explanation of these substantial differences. Differences in recording behaviour probably play a small role in the explanation of the variation in prescription of PPCM between FPs, because a better or worse recording behaviour will influence the numerator and denominator in a similar way.

For all four patient groups there is a considerable unexplained variation that justifies further research to investigate why these differences exist, and perhaps more importantly to get insight into the impact of differences in prescribing behaviour on CVD morbidity and mortality for these four patient groups. Positive (i.e. high prescribing behaviour of cardiovascular medication results in higher survival rates and less adverse effects) as well as negative (no difference in health outcomes between FPs with a high or a low prescribing behaviour of cardiovascular medication) outcomes are relevant. A positive outcome justifies the expenditure on cardiovascular medication from a societal perspective. A negative outcome indicates that important cost savings may be achieved by reassessing the prescription of cardiovascular medication, because a relatively low prescribing behaviour of cardiovascular medication may achieve similar health outcomes compared with a relatively high prescribing behaviour. For the same reason, further research is recommended to investigate whether higher spending on cardiovascular preventive activities reduces prescribing and vice a versa.

Differences in PPCM prescribing behaviour between FPs that is not explained by patient's or practice's characteristics may indicate under-treatment. From a patient-care perspective it is important to have insight into these differences. Under-treatment justifies an increase in resources, whereas over-treatment indicates inefficiency. Another relevant implication for clinical practice is that our research shows that the current guideline is interpreted differently. Improvement of the current guideline may be useful, especially for primary prevention, for which the discrepancy between family practices is considerable.

Our study has some limitations. First, although we identified primary preventive activities for patients with unrecognized cardiovascular disease from literature and interviews with general practitioners, this careful procedure does not guarantee that activities we did not include are irrelevant. Second, we assumed that data from the questionnaire reliably indicated the true extent of primary preventive activities. By definition, data taken from self-administered questionnaires are estimations. However, the FPs who filled in the questionnaire belong to the Netherlands Information Network of General Practice, were representative for all Dutch FPs, and had experience with filling in self-complete questionnaires. Therefore, we believe that the estimates based on the questionnaire are not that far from reality. Third, we assumed that all patients with a history of CVD should have contacted their general practitioner at least once for cardiovascular reasons in 2005-2007. As a result some patients with a history of CVD that did not contact their general practitioner for cardiovascular reasons in 2005-2007 were regarded as primary preventive nature. As this latter group is small, we do not expect that the reported results are changed considerably. Fourth, although we took into account different factors that might have an impact on variations in prescribing (e.g., age, gender, neighbourhood), there may be other factors for which we did not account for such as ethnicity and marital status. Fifth, we defined expenditure on medication as those spent on statins and beta blockers. However, other (primary) preventive cardiovascular medication exists. Aspirin, for example, can be used as a (primary) preventive cardiovascular medication as well. However, this medication is also used as an analgesic to relieve minor aches and pains, as an antipyretic to reduce fever, and as an anti-inflammatory medication. Therefore for practical reasons we focused on statins and beta blockers only. The costs of total spending on primary preventive cardiovascular medication may therefore be an underestimation. Sixth, to investigate the prescription of primary preventive cardiovascular medication in FPs, we had to include FPs with complete data sets over the whole period 2005-2007. From the FPs with complete data sets, the FPs' characteristics (practice type, urbanisation) were quite similar to the Dutch national situation of FPs. Nevertheless, we cannot rule out that FPs with incomplete data sets may have different prescribing behaviour than FPs with incomplete data sets. Finally, our results may have been influenced by specific characteristics of the Dutch health care system, in which the family practice plays a pivotal role. Having said this, we still think our study has a wider applicability because medical procedures used for the prevention and treatment of cardiovascular diseases in the Netherlands are very similar to those in other developed countries.

## Conclusions

This study provides important new insights regarding the costs of cardiovascular primary preventive activities, which is crucial for health policy development and evaluation. Although the frequency of cardiovascular primary preventive activities such as blood pressure measurements and lifestyle counselling in FPs are sizeable, total costs of these activities are relatively low compared to the costs of primary preventive cardiovascular medication (PPCM) prescribed by general practitioners. Further research is needed to determine whether these relatively high costs of PPCM are justified as there is a considerable heterogeneity in prescribing behaviour of PPCM between FPs. Striving for an optimal use of cardiovascular primary preventive activities might lead to similar health outcomes, but may achieve important cost savings.

## Competing interests

The authors declare that they have no competing interests.

## Authors' contributions

EWBG developed the study protocol, performed the study, analyzed the data and wrote the manuscript. LCJS and MB initiated and supervised the study, and developed the study protocol. SD and RAV coordinated and contributed to the data collection. All authors read and approved the final manuscript.

## Pre-publication history

The pre-publication history for this paper can be accessed here:

http://www.biomedcentral.com/1471-2296/12/69/prepub

## Supplementary Material

Additional File 1 **Appendix**. Time spent on primary preventive activities in family practice to prevent cardiovascular diseases in the Netherlands in 2009, in minutes per week per discipline. Click here for file
