# Supplementary material for: Primary prevention of cardiovascular diseases: a cost study in family practices
Source: BMC Fam Pract. 2011 Jul 6;12:69. doi: 10.1186/1471-2296-12-69 (PMC3160896; doi:10.1186/1471-2296-12-69)
Supplement: Additional File 1 — Appendix. Time spent on primary preventive activities in family practice to prevent cardiovascular diseases in the Netherlands in 2009, in minutes per week per discipline. [file 1471-2296-12-69-S1.DOC]

**Appendix**

**appendix 1:** Time spent on primary preventive activities in family practice to prevent cardiovascular diseases in the Netherlands in 2009, in minutes per week per discipline.
